# Emergency Departments’ Uptake of Telehealth for Stroke Versus Pediatric Care: Observational Study

**DOI:** 10.2196/33981

**Published:** 2022-06-20

**Authors:** Kori S Zachrison, Emily M Hayden, Krislyn M Boggs, Tehnaz P Boyle, Jingya Gao, Margaret E Samuels-Kalow, James P Marcin, Carlos A Camargo Jr

**Affiliations:** 1 Department of Emergency Medicine Massachusetts General Hospital Boston, MA United States; 2 Department of Pediatrics Boston Medical Center Boston, MA United States; 3 Department of Pediatrics University of California Davis School of Medicine University of California Sacramento, CA United States

**Keywords:** telehealth, telemedicine, emergency care, stroke, pediatric care

## Abstract

**Background:**

Telehealth for emergency stroke care delivery (telestroke) has had widespread adoption, enabling many hospitals to obtain stroke center certification. Telehealth for pediatric emergency care has been less widely adopted.

**Objective:**

Our primary objective was to determine whether differences in policy or certification requirements contributed to differential uptake of telestroke versus pediatric telehealth. We hypothesized that differences in financial incentives, based on differences in patient volume, prehospital routing policy, and certification requirements, contributed to differential emergency department (ED) adoption of telestroke versus pediatric telehealth.

**Methods:**

We used the 2016 National Emergency Department Inventory–USA to identify EDs that were using telestroke and pediatric telehealth services. We surveyed all EDs using pediatric telehealth services (n=339) and a convenience sample of the 1758 EDs with telestroke services (n=366). The surveys characterized ED staffing, transfer patterns, reasons for adoption, and frequency of use. We used bivariate comparisons to examine differences in reasons for adoption and use between EDs with only telestroke services, only pediatric telehealth services, or both.

**Results:**

Of the 442 EDs surveyed, 378 (85.5%) indicated use of telestroke, pediatric telehealth, or both. EDs with both services were smaller in bed size, volume, and ED attending coverage than those with only telestroke services or only pediatric telehealth services. EDs with telestroke services reported more frequent use, overall, than EDs with pediatric telehealth services: 14.1% (45/320) of EDs with telestroke services reported weekly use versus 2.9% (8/272) of EDs with pediatric telehealth services (*P*<.001). In addition, 37 out of 272 (13.6%) EDs with pediatric telehealth services reported no consults in the past year. Across applications, the most frequently selected reason for adoption was “improving level of clinical care.” Policy-related reasons (ie, for compliance with outside certification or standards or for improving ED performance on quality metrics) were rarely indicated as the most important, but these reasons were indicated slightly more often for telestroke adoption (12/320, 3.8%) than for pediatric telehealth adoption (1/272, 0.4%; *P*=.003).

**Conclusions:**

In 2016, more US EDs had telestroke services than pediatric telehealth services; among EDs with the technology, consults were more frequently made for stroke than for pediatric patients. The most frequently indicated reason for adoption among all EDs was related to clinical care.

## Introduction

Resource availability in US emergency departments (EDs) varies substantially, with major disparities in access to specialists [1]. With increasing regionalization of care, consultants may become less available in smaller or more rural EDs [2,3]. Telehealth has been increasingly acknowledged as a tool that may mitigate these disparities in access. With rapid expansion in the use of telehealth in emergency care delivery during the COVID-19 pandemic [4-6], it is possible that growth in technological infrastructure may be harnessed for longer-term solutions.

Telehealth for emergency stroke care delivery (telestroke) has a history of successful implementation, with a large body of work demonstrating improved delivery of stroke care [7-10]. One possible explanation for the extensive adoption of telestroke may be that it can provide a more cost-effective way for hospitals to achieve certification requirements that would have otherwise been difficult, if not impossible, to attain. For example, by providing 24/7 access to neurology consultation, telestroke has likely enabled many hospitals to achieve stroke center status without the expense of fully staffing in-person neurologist coverage. By achieving this designation, hospitals may then advertise themselves as stroke centers, may receive more stroke patient transports from prehospital emergency medical services (EMS), and may have the ability to admit stroke patients who may have otherwise been transferred to another facility. Given the generally favorable billing associated with diagnosis-related groups (DRGs) for patients admitted with stroke diagnoses, hospitals may readily see the financial advantages of investing in telestroke services.

In contrast to stroke, telehealth in pediatric emergency care (ie, pediatric telehealth) is infrequently used [11]. Many studies have demonstrated the relationship between telehealth and improved care and decision-making in the care of critically ill children in rural EDs [12,13], to help avoid unnecessary transfers [14], and to improve patient satisfaction [12]. Yet even when pediatric telehealth programs exist, low consult volumes often lead to discontinuation [15]. While designations for pediatric EDs exist, unlike stroke center certification, there is not a widely advertised national pediatric emergency care certification program that would enable hospitals to tout their certification status or to admit patients with advantageous billing. Overall, the financial and policy incentives for telestroke adoption are largely absent with respect to pediatric telehealth. We hypothesized that this contrast between stroke and pediatric emergency care might be an important factor driving differential uptake of telehealth for stroke versus for pediatric care.

To better understand barriers and motivators of telehealth adoption in EDs, we surveyed US EDs with telestroke and pediatric telehealth prior to the COVID-19 pandemic. Our primary objective was to determine whether differences in policy or certification requirements contributed to differential uptake of telestroke versus pediatric telehealth. We hypothesized that differences in hospital financial incentives, based on differences in patient volume, prehospital patient routing policy, and certification requirements, contributed to differential ED adoption of telestroke versus pediatric telehealth. These findings may have implications for health system leaders or policy makers interested in increasing uptake of pediatric telehealth.

## Methods

### Study Design, Selection of Participants, Survey, and Administration

We used data from the 2016 National Emergency Department Inventory–USA (NEDI-USA) survey responses to classify all responding EDs based on use of telehealth for stroke and for pediatric emergency care, and we targeted these EDs for a follow-up survey. The NEDI-USA survey is a brief, one-page survey that collects basic ED characteristics, including staffing and telehealth use, from EDs nationally (n=5404). The survey was administered in 2017 to characterize US EDs in 2016. The NEDI-USA survey is included in Multimedia Appendix 1 and was coordinated by the Emergency Medicine Network (EMNet) [16]; methods have been previously reported, including details of the telehealth component of the survey [11]. In 2018, as part of a study focused on understanding barriers and facilitators to ED adoption of telehealth, we administered a set of follow-up surveys to EDs using telehealth for stroke and for pediatric emergency care; this was done to understand the differential motivators of telehealth adoption between EDs using telehealth for stroke versus for pediatric emergency care. The surveys characterized details of the ED and clinical care, barriers to use for nonusers, and details of telehealth use in the preceding year for users.

Based on our a priori sample size calculations, we determined that we would need 453 EDs with telestroke services and 453 EDs with pediatric telehealth services for our follow-up survey in order to detect a 10% difference in the proportion of EDs indicating a policy-based motivation for adoption, assuming an α value of .05 and power of 0.80. There were more than 453 EDs with telestroke services (n=1758) but fewer than 453 EDs with pediatric telehealth services (n=339). Among these, there were 259 EDs that reported both pediatric telehealth and telestroke services. We identified a random sample of 366 EDs with telestroke services but not pediatric telehealth services, and all EDs that reported only pediatric telehealth services (n=339). Thus, the final population of EDs receiving the second survey included 76 EDs with pediatric telehealth but not telestroke services, 263 EDs with telestroke and pediatric telehealth services, and 103 EDs with telestroke but not pediatric telehealth services; this generated a total of 442 EDs for the follow-up survey. This study was conducted as part of a larger grant-funded study with other aims, related to understanding barriers and facilitators of ED adoption and use of telehealth [17,18]; this included a separate survey to rural EDs that did not receive telestroke or pediatric telehealth certification. On that survey, some responding EDs subsequently clarified that they did have telestroke or pediatric telehealth services; these EDs were then included in this analysis.

The follow-up survey varied slightly based on the nature of telehealth use in the surveyed EDs. The survey included additional questions characterizing ED staffing and transfer patterns, as these may influence telehealth adoption; provider perceptions of reasons for telehealth adoption; and estimated frequency of telehealth use. This survey included a combination of questions from prior research [19], as well as questions specifically developed for the aims of this study. The newly added questions were developed with input from several telehealth researchers, as well as emergency medicine researchers and nonresearch faculty. An example of the version of the survey including questions for EDs with telestroke and pediatric telehealth services is included in Multimedia Appendix 1.

We mailed the follow-up surveys by post to ED directors twice over a 3-month period and included a link to a web-based version of the survey in each mailing. We also followed up with nonresponsive and partially responding sites via telephone. Survey data were managed using REDCap (Research Electronic Data Capture; Vanderbilt University).

### Outcomes

**Figure 1:**
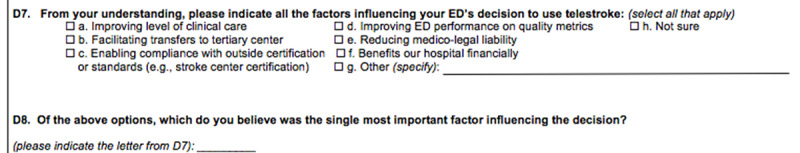
Screenshot of the survey question regarding the reason for telehealth adoption. ED: emergency department.

The primary outcome was reason for telehealth adoption. We dichotomized responses as motivated by policy or certification requirements (“yes” or ”no”). This was based on the response to the question asking about the single most important factor influencing the decision to adopt telehealth (Figure 1). Response options included (1) improving level of clinical care, (2) facilitating transfers to tertiary center, (3) enabling compliance with outside certification or standards, (4) improving ED performance on quality metrics, (5) reducing medicolegal liability, (6) benefits our hospital financially, (7) other (specify), and (8) not sure. Responses classified as policy or certification motivated were “enabling compliance with outside certification or standards” and “improving ED performance on quality metrics.” Any free-text responses included in the “other” section were independently reviewed and coded by two authors (KSZ and EMH) as policy or certification motivated or not.

### Other Variables of Interest

The full survey is included in Multimedia Appendix 1. We also collected data on ED volume and characteristics of the ED space and staffing. We collected stroke-related variables, including certification status, typical treatment and stroke patient dispositions, availability of neurologists, and frequency of telestroke use in 2016. We collected pediatric emergency care–related variables, including who typically cares for a child presenting to the ED, availability of in-person pediatric consultation, and estimated frequency of pediatric telehealth consultation in 2016.

We identified academic EDs as those that were the primary site for an emergency medicine residency [20]. We identified rural EDs as those located outside of a core-based statistical area [21]. We used data from the Center for Connected Health Policy [22] and the American Telehealth Association 2016 Gaps Analysis [23] to identify states’ telehealth policy environment based on state policy in 2016. States were categorized as having no coverage parity (ie, no requirement for payors to reimburse telehealth care), a partial or conditional mandate for payment parity, or full payment parity.

### Analysis

Data analysis was performed using SAS software (version 9.4; SAS Institute Inc). Our analysis focused on EDs indicating that they had telestroke services, pediatric telehealth services, or both in 2016. We compared EDs by telehealth usage using the Kruskal-Wallis test for continuous variables, the chi-square test for categorical variables, and the Fisher exact test for small-sized categorical variables of interest (ie, >20% of cells with expected frequencies of <5). For simplicity, we report *P* values only for the key comparisons. We addressed our research hypothesis by determining the proportion of telestroke EDs for which the reason for adoption was policy motivated, and the proportion of pediatric telehealth EDs for which the reason for adoption was policy motivated.

### Ethics Approval

This study was approved by the Mass General Brigham Institutional Review Board (protocol No. 2017P000130).

## Results

### Overview

The 2016 NEDI-USA survey yielded responses from 4506 out of 5404 (83.38%) EDs; 4410 out of 5404 (81.61%) EDs responded to the telehealth question asking them to report presence or absence of telehealth in the ED [11]. Based on the responses to the telehealth questions on the NEDI-USA, we identified EDs using telestroke and pediatric telehealth for our follow-up survey. Details of the sampling strategy are included in the Methods.

Of the 442 EDs sampled for our follow-up survey, 378 (85.5%) responded; this included 106 (28.0%) EDs with telestroke but not pediatric telehealth, 214 (56.6%) EDs with telestroke and pediatric telehealth, and 58 (15.3%) EDs with pediatric telehealth but not telestroke.

### ED Characteristics

Characteristics of the 378 EDs in our sample are provided in Table 1. EDs had a median annual volume of 9959 (IQR 2475-30,000) visits and a median annual pediatric volume of 1800 (IQR 429-5163) visits. Very few were academic EDs (n=6, 1.6%), but nearly half were rural (n=179, 47.4%). More EDs were in the Midwest (n=144, 38.1%) and the South (n=102, 27.0%) than in the West (n=79, 20.9%) and the Northeast (n=49, 13.0%). Most EDs were in states without any payment parity policy (n=237, 62.7%), though 107 (28.3%) were in states with partial payment parity, and 34 (9.0%) were in states with full payment parity. Other frequently reported applications of telehealth in these EDs included psychiatry (n=173, 45.8%) and trauma (n=159, 42.1%).

**Table 1:** Emergency department characteristics overall and by type of telehealth used.

ED^a^ characteristics		All EDs in sample (N=378)	EDs with telestroke only (n=106)	EDs with telestroke and pediatric telehealth (n=214)	EDs with pediatric telehealth only (n=58)
ED volume (visits), median (IQR)		9959 (2475-30,000)	20,945 (8605-40,033)	4783 (1278-20,000)	14,733 (6005-35,400)
ED pediatric volume (visits), median (IQR)		1800 (429-5163)	3664 (1596-6590)	860 (240-3211)	2993 (766-6000)
Pediatric space in ED, n (%)		39 (10.3)	15 (14.2)	15 (7.0)	9 (15.5)
PECC^b^, n (%)		46 (12.1)	13 (12.3)	23 (10.7)	10 (17.2)
Total number of beds (adult and pediatric), median (IQR)		9 (4-19)	13 (8-24)	6 (3-14)	12 (6-20)
Number of FTE^c^ attendings, median (IQR)		4 (2-8)	6 (4-12)	4 (1-6)	5 (4-8)
**Proportion of attending emergency physicians BC/BE^d^ by ABEM^e^, AOBEM^f^, or ABP^g^ in pediatric emergency medicine (%), n (%) **					
	<20	104 (27.5)	21 (19.8)	71 (33.2)	12 (20.7)
	20-49	32 (8.5)	10 (9.4)	16 (7.5)	6 (10.3)
	50-79	34 (9.0)	7 (6.6)	17 (7.9)	10 (17.2)
	80-100	156 (41.3)	55 (49.1)	77(36.0)	24 (41.4)
	Missing	52 (13.8)	13 (12.3)	33 (15.4)	6 (10.3)
Academic, n (%)		6 (1.6)	3 (2.8)	1 (0.5)	2 (3.4)
Rural location, n (%)		179 (47.4)	32 (30.2)	125 (58.4)	22 (37.9)
**Region, n (%)**					
	Northeast	51 (13.5)	17 (16.0)	18 (8.4)	16 (27.6)
	Midwest	143 (37.8)	30 (28.3)	101 (47.2)	12 (20.7)
	South	103 (27.2)	37 (34.9)	46 (21.5)	20 (34.5)
	West	81 (21.1)	22 (20.8)	49 (22.8)	10 (17.2)
**State payment policy, n (%)**					
	Full parity	34 (9.0)	6 (5.7)	21 (9.8)	7 (12.1)
	Partial parity	107 (28.3)	35 (33.0)	52 (24.3)	20 (34.5)
	None	237 (62.7)	65 (61.3)	141 (65.9)	31 (53.4)
**Other specialties for which ED receives telehealth, n (%)**					
	Psychiatry	173 (45.8)	30 (28.3)	125 (58.4)	18 (31.0)
	Trauma	159 (42.1)	21 (19.8)	126 (58.9)	12 (20.7)
	Dermatology	54 (14.3)	5 (4.7)	44 (20.6)	5 (8.6)
	Radiology	59 (15.6)	8 (7.5)	48 (22.5)	3 (5.2)

^a^ED: emergency department.

^b^PECC: pediatric emergency care coordinator.

^c^FTE: full-time equivalent.

^d^BC/BE: board certified or board eligible.

^e^ABEM: American Board of Emergency Medicine.

^f^AOBEM: American Osteopathic Board of Emergency Medicine.

^g^ABP: American Board of Pediatrics.

### Stroke Care and Telestroke Use

EDs with telestroke services only were more frequently Joint Commission–certified stroke centers relative to those with telestroke and pediatric telehealth services or pediatric telehealth services only (Table 2). With respect to availability of an in-person neurologist, EDs with telestroke and pediatric telehealth had the least availability. Of all telestroke EDs (n=320), 45 (14.1%) reported weekly use and another 44 (13.8%) reported using telestroke services every 1 to 2 weeks during 2016. Fewer than one-third of EDs with telestroke reported administering alteplase without a telestroke consultation (n=94, 29.4%). There were no significant differences in admission practices between groups, with the exception of admission of alteplase-treated patients. EDs with only telestroke services reported capacity to admit alteplase-treated stroke patients more frequently (36/106, 34.0%) relative to EDs with both telestroke and pediatric telehealth services (42/214, 19.6%) or EDs with only pediatric telehealth services (13/58, 22%; *P*=.02).

**Table 2:** Telestroke use and the clinical care of stroke patients.

ED^a^ characteristics			All EDs in sample (N=378), n (%)	EDs with telestroke only (n=106), n (%)			EDs with telestroke and pediatric telehealth (n=214), n (%)			EDs with pediatric telehealth only (n=58), n (%)		
Joint Commission certification			117 (31.0)	45 (42.5)			56 (26.2)			16 (27.6)		
If no Joint Commission certification, alternative stroke certification status			59/261 (22.6)	16/61 (26.2)			37/158 (23.4)			6/42 (14.3)		
Neurologist available in person in the ED			63 (16.7)	27 (25.5)			25 (11.7)			11 (19.0)		
**If neurologist available in person, timing of arrival (minutes)^b^**												
	0-29	39 (61.9)			18 (66.7)			14 (56.0)			7 (63.6)	
	30-59	16 (25.4)			8 (29.6)			5 (20.0)			3 (27.3)	
	≥60	5 (7.9)			0 (0)			4 (16.0)			1 (9.1)	
If neurologist available, is available 24/7^b^			38 (60.3)			17 (63.0)			12 (48.0)			9 (81.8)
**Approximate number of telestroke consultations in 2016**												
	None	37 (9.8)			10 (9.4)			27 (12.6)			N/A^c^	
	<12 (<1/month)	133 (35.2)			33 (31.1)			100 (46.7)			N/A	
	12-25 (every 3-4 weeks)	41 (10.8)			18 (17.0)			23 (10.7)			N/A	
	26-52 (every 1-2 weeks)	44 (11.6)			15 (14.2)			29 (13.6)			N/A	
	>52 (>1/week)	45 (11.9)			22 (20.8)			23 (10.7)			N/A	
	Missing	20 (5.3)			8 (7.5)			12 (5.6)			N/A	
**In 2016, was alteplase ever administered to a stroke patient in the ED without a telestroke consultation?**												
	Yes	94 (24.9)			30 (28.3)			64 (30.0)			N/A	
	No	167 (44.2)			55 (51.9)			112 (52.3)			N/A	
	Not sure	46 (12.2)			16 (15.1)			30 (14.0)			N/A	
**In 2016, approximately how many stroke patients were treated with alteplase in your ED?**												
	0	29 (7.7)			5 (4.7)			21 (9.8)			3 (5.0)	
	1-3	129 (34.1)			28 (26.4)			83 (38.8)			18 (31.0)	
	≥4	172 (49.4)			56 (52.8)			84 (39.3)			32 (55.2)	
	Not sure	36 (9.5)			14 (13.2)			20 (9.3)			2 (3.4)	
**Patients typically admitted by hospital**												
	Patients who experienced TIA^d^	292 (77.2)			85 (80.2)			164 (76.6)			43 (74.1)	
	Patients who experienced stroke, without alteplase	215 (56.9)			64 (60.4)			113 (52.8)			38 (65.5)	
	Patients who experienced stroke, treated with alteplase	91 (24.1)			36 (34.0)			42 (19.6)			13 (22.4)	

^a^ED: emergency department.

^b^These values are based on the number of neurologists available in person in the ED—all EDs: n=63; EDs with telestroke: n=27; EDs with both: n=25; EDs with pediatric telehealth: n=11.

^c^N/A: not applicable; no telestroke services.

^d^TIA: transient ischemic attack.

### Pediatric Care and Pediatric Telehealth Use

The vast majority of EDs in the sample reported that children were generally cared for by a general emergency physician (289/378, 76.5% overall); however, this did vary by category of telehealth use (Table 3). Among all EDs with pediatric telehealth services, frequency of use was relatively low, with 8 out of 272 (2.9%) reporting weekly use, and 12 (4.4%) reporting use every 1 to 2 weeks. Most (164/272, 60.3%) reported use fewer than 12 times over the year. Many EDs with pediatric telehealth did report using telehealth for pediatric mental health consultation (82/272, 30.1%).

**Table 3:** Use of telehealth for pediatric emergency care and the clinical care of children.

ED^a^ characteristics			All EDs in sample (N=378), n (%)		EDs with telestroke only (n=106), n (%)		EDs with telestroke and pediatric telehealth (n=214), n (%)		EDs with pediatric telehealth only (n=58), n (%)
**Who typically cares for a child presenting to the ED at 6 PM on a typical day?**									
	Pediatric emergency physician	19 (5.0)		8 (7.5)		6 (2.8)		5 (8.6)	
	General emergency physician	289 (76.5)		94 (88.7)		145 (67.8)		50 (86.2)	
	General pediatrician	23 (6.1)		9 (8.5)		9 (4.2)		5 (8.6)	
	Physician of another specialty	90 (23.8)		17 (16.0)		62 (29.0)		11 (19.0)	
	Physician assistant or nurse practitioner	252 (66.7)		70 (66.0)		148 (69.2)		34 (58.6)	
**Professional available for in-person pediatric consultation**									
	Pediatrics attending	109 (28.8)		45 (42.4)		43 (20.1)		21 (36.2)	
	Pediatrics trainee	9 (2.4)		3 (2.8)		4 (1.9)		2 (3.4)	
	Family medicine attending	128 (33.9)		34 (32.1)		76 (35.5)		18 (31.0)	
	Family medicine trainee	11 (2.9)		3 (2.8)		5 (2.3)		3 (5.2)	
	Other	40 (10.6)		10 (9.4)		24 (11.2)		6 (10.3)	
	None	144 (38.1)		32 (30.2)		92 (43.0)		20 (34.5)	
Does a physician assistant or nurse practitioner ever provide care for a child in the ED? (yes)			283 (74.9)		77 (72.6)		164 (76.6)		42 (72.4)
If yes to above, are they supervised by the on-site attending? (yes)			174/283 (61.5)		56/77 (72.7)		81/164 (49.4)		37/42 (88.1)
**In 2016, approximate number of telehealth consultations for pediatric emergency care**									
	None	37 (9.8)		N/A^b^		29 (13.6)		8 (13.8)	
	<12 (<1/month)	164 (43.4)		N/A		127 (59.2)		37 (63.8)	
	12-25 (every 3-4 weeks)	40 (10.6)		N/A		32 (15.0)		8 (13.8)	
	26-52 (every 1-2 weeks)	12 (3.2)		N/A		10 (4.7)		2 (3.5)	
	>52 (>1/week)	8 (2.1)		N/A		8 (3.7)		0 (0)	
In 2016, did your ED ever use telehealth for pediatric mental health consultation? (yes)			82 (21.7)		N/A		69 (32.2)		13 (22.4)

^a^ED: emergency department.

^b^N/A: not applicable; no pediatric telehealth services.

### Policy- Versus Nonpolicy-Motivated Adoption of Telehealth

Among all EDs with telestroke services, 213 out of 320 (66.6%) reported a policy-motivated reason for adoption, whereas among EDs with pediatric telehealth services, 138 out of 272 (50.7%) did so (Table 4). When asked to select the *single most important* factor influencing the decision for adoption, policy-motivated reasons were rarely selected, but they were selected slightly more frequently by EDs with telestroke services (12/320, 3.8%) than by EDs with pediatric telehealth services (1/272, 0.4%; *P*=.003). Reasons specified when “other” was selected are included in Table S1 in Multimedia Appendix 1.

**Table 4:** Factors influencing emergency department use of telehealth.

Factor	EDs^a^ selecting factors influencing use of telestroke (n=320), n (%)		EDs selecting factors influencing use of pediatric telehealth (n=272)		
	When selecting *all that apply*	When selecting the s*ingle most important* factor	When selecting *all that apply*	When selecting the s*ingle most important* factor	
Improving level of clinical care	276 (86.3)	223 (69.7)	231 (84.9)	187 (68.8)	
Facilitating transfer to tertiary center	236 (73.8)	34 (10.6)	218 (80.1)	56 (20.6)	
Enabling compliance with outside certification or standards	141 (44.1)	5 (1.6)	75 (27.6)	0 (0)	
Improving ED performance on quality metrics	198 (61.9)	7 (2.2)	128 (47.1)	1 (0.4)	
Reducing medicolegal liability	136 (42.5)	2 (0.6)	107 (39.3)	2 (0.7)	
Benefits our hospital financially	57 (17.8)	2 (0.6)	50 (18.4)	1 (0.4)	
Other	27 (8.4)	18 (5.6)	20 (7.4)	8 (2.9)	
Not sure	11 (3.4)	11 (3.4)	4 (1.5)	3 (1.1)	
Missing	15 (4.7)	18 (5.6)	13 (4.8)	14 (5.1)	
Policy motivated^b^	213 (66.6)	12 (3.8)	138 (50.7)	1 (0.4)	

^a^ED: emergency department.

^b^This response was based on the following two responses: “enabling compliance with outside certification or standards” and “improving ED performance on quality metrics.”

## Discussion

### Principal Findings

In this study, we surveyed a national sample of EDs with telestroke services and all EDs with pediatric telehealth services prior to the COVID-19 pandemic. Among these EDs, whether using telehealth for telestroke, pediatric telehealth, or both, the single most commonly reported factor driving telehealth use was for the purpose of improving clinical care. Policy- or certification-related reasons were selected as a motivator by many EDs, more often for telestroke services than for pediatric telehealth services. However, when asked about the single most important reason, the vast majority of all EDs indicated that telehealth was used for improving clinical care.

### Comparison to Prior Work

There has been little previous work focusing specifically on EDs’ reasons for adoption of particular lines of telehealth services. A previous mixed methods study of 17 programs providing pediatric telehealth services reported a number of barriers and facilitators to adoption and successful maintenance of telehealth programs [15]. The investigators suggested that particular policy-related solutions may be effective for realigning incentives and enabling more widespread adoption. One suggested solution is particularly underscored by our results. Specifically, the investigators found that insufficient consult volume was a problem that contributed to program closure, and noted that in the setting of inadequate volume it may be difficult to maintain competency with technology and may also be difficult to justify the investment [15]. Likewise, we also found that EDs with pediatric telehealth services reported infrequent use in the majority of cases, with 77% of these EDs reporting use that was less than one time per month, on average, during the previous year. This contrasted with EDs with telestroke services where fewer than half reported such infrequent use. It is not surprising that suspected strokes are more common than sick children requiring telehealth consultation. Further, the framework of the technology acceptance model points to perceived usefulness as an important driver of telehealth’s acceptance [24]. However, it is the very nature of the rarity of a critically ill pediatric patient that makes telehealth such a potentially effective tool. If an emergency physician in a relatively low-volume ED sees a critically ill child as an exceedingly rare event, then having the ability to connect with an expert consultant becomes that much more valuable. This is particularly true given that many EDs have been found to have critical deficiencies in pediatric emergency services [25-27]. Benefits may also be realized in the use of pediatric telehealth for less critically ill children. One recent study demonstrated a successful pediatric telehealth program in which the implementation was supported, adoption and use increased over time, and efficiency of health care resource use improved [28]. Our surveys did not capture how many times an ED reached out to either a pediatric critical care physician or a pediatric emergency medicine physician outside of a formal telehealth program. It may be possible in pediatric telehealth that EDs may not feel that the volume of sick children is enough for a pediatric telehealth subscription. It is likely that there was less of a desire to subscribe to such a program if prior to such a program offering they were able to connect with well-meaning pediatric acute care specialists who would advise the ED provider on the management of the patient over the phone.

### Implications and Future Directions

Despite compelling examples of successful pediatric telehealth programs [13,29] and the endorsement of pediatric telehealth by the National Academy of Medicine as a solution to address disparities in access to care [30], our results underscore the relatively infrequent use of pediatric telehealth services relative to telestroke services by EDs nationally prior to the COVID-19 pandemic. In 2016, only 339 EDs reported having pediatric telehealth services, as compared to 1758 EDs with telestroke services. We had hypothesized that the ability to obtain external certification or to improve performance on national quality metrics for stroke may have been an important driver in the significantly higher prevalence of telestroke services as compared to pediatric telehealth services. However, when examining the single most important reason for adoption, our results do not fully support that hypothesis. It may be that ED directors were not the appropriate source of this information and that a hospital-level financial administrator may have had more insight into the decision. An alternative explanation may be related to the nature of the typical telehealth consult for these conditions. For example, the typically envisioned telestroke consultation may be a patient with a potential stroke and an emergency physician looking for additional guidance, expertise, or shared liability in the decision to treat with thrombolytic therapy. In contrast, in the setting of a critically ill child in a remote ED, the emergency physician is often hoping to transfer the child as quickly as possible, rather than to delay transfer with a telehealth consultation [15]. In particular, given that small EDs may not have the ability to admit children to their hospital, many have no choice but to transfer these pediatric patients, and a pediatric consultation may be considered of lower value if patients are inevitably transferred.

In addition to differences in ED clinical care, the comparison of telestroke to pediatric telehealth services in EDs is an “apples-to-oranges” comparison in other ways as well. Indeed, these differences are an important part of what interested us in the comparison and in better understanding differential drivers of adoption. This includes differences in volume, in prehospital considerations, and in hospital financial motivations. Whereas EDs likely see consistent volumes of patients with stroke-like symptoms, a critically ill pediatric patient is a much more infrequent event, and such differences in patient volume may be an important factor contributing to different perceptions of the need for telestroke services as compared to pediatric telehealth services. Prehospital EMS patient triage may also contribute. An ED that requires telehealth services to connect with pediatric expertise would likely prefer that a critically ill child be transported directly to a higher-resourced center when possible. In contrast, prehospital considerations for patients with suspected stroke are much different. The time-dependent benefit of acute stroke interventions means that the closest capable hospital is considered the optimal transport destination [31]. Prehospital stroke triage policies vary by region; however, typically, in order to be considered a “capable” hospital, an ED needs to have access to neurologist expertise, generally reflected as stroke center certification. Often, the most cost-effective way to achieve 24/7 neurologist access and stroke center certification is through a telestroke program. In addition, while EDs may also receive a pediatric readiness score [26,32,33], this is not a certification process, per se. Some states do have their own pediatric emergency facility recognition programs [34,35]; however, these are not universal and we did not explicitly study whether the existence of these standards motivated pediatric telehealth adoption. Finally, once the patient with suspected stroke is transported to an ED, the hospital continues to have financial incentives to admit the patient rather than to transfer, as DRG billing for stroke care is generally favorable.

These differences in patient volume, in prehospital triage decisions, and in hospital financial incentives for disposition decisions were what motivated our research question and hypothesis. Nevertheless, they are also important reasons for telehealth adoption that our approach did not fully capture. Given previous research showing that cost was a major barrier for EDs without telehealth services [17], these differences in hospitals’ anticipated return on investment for telestroke versus pediatric telehealth programs are important. One potential solution for these smaller or lower-volume EDs is to capitalize on economies of scale to facilitate implementation of telehealth. If the technology can be in place and shared among various applications, then the expense of implementation may be more justifiable, and the providers may be more able to maintain a baseline level of comfort and competence with the technology as well. Indeed, we found that relative to EDs with telestroke services alone or pediatric telehealth services alone, those EDs with both telestroke and pediatric telehealth services were smaller, lower-volume sites with fewer full-time equivalent attendings on staff. It may be that these economies of scale are already being realized by these EDs. This may be a good target for future research or future grant programs for smaller or less resourced hospitals.

Finally, in our recent experience during the COVID-19 pandemic, we have seen tremendous growth in the use of telehealth in health care, specifically emergency medicine [4-6]. Many of these changes were stimulated by clinical need in combination with changes in telehealth legislation and reimbursement policy. This highlights the interconnected relationship in which clinical need and policy changes worked together to increase adoption and use of the technology. This also underscores the importance of understanding drivers of telehealth adoption and use moving forward. By characterizing barriers and facilitators of telehealth in emergency care and understanding how these factors vary between clinical indications, we may be better equipped to ensure that EDs that benefit from the technology are able to continue to use telehealth to provide optimal clinical care. Future qualitative work may be of particular value to better understand these motivators.

### Limitations

Our results predate the dramatic changes in telehealth that occurred during the pandemic and are likely not representative of current use or of what lies ahead. However, in many ways, the COVID-19 experience has generated new equivalents for the “geographic distance” that telehealth was previously bridging. With new limitations to access in more urban settings as well as clinical demands related to the pandemic, it will continue to be important to understand drivers of and barriers to adoption of telehealth use in emergency care. We believe that our findings remain relevant to that question. Our study has other limitations as well. Our results were self-reported by ED directors, and these individuals may not have had full insight into their hospitals’ decisions to implement telehealth technology; a survey targeted to a group with a different role may have produced different results. Survey responders may also have been confused about the definition of telehealth. While we were able to confirm and clarify this with the EDs responding by phone, those EDs responding by postal mail did not have this opportunity. It is also possible that our results were subject to selection bias related to nonresponders. However, response rates for our survey were generally high, and the characteristics of responding and nonresponding EDs were similar (Table S2 in Multimedia Appendix 1). It is also worth noting that EDs in our sample had a variety of staffing models, and the nature of pediatric telehealth use may vary based on the training of the clinician seeing the patient. For example, most emergency and family physicians are comfortable with routine pediatric conditions; however, physician assistants, nurse practitioners, or adult-trained physicians may be less so. Future work may further explore these differences. Finally, by asking about the single most important factor driving telehealth adoption, we cannot fully comment on whether policy is an important driver to motivate adoption. It may be necessary but not sufficient, or it may not be considered the single most important factor when the other response options also include “improving clinical care.”

### Conclusions

Prior to the COVID-19 pandemic, pediatric telehealth services were less common than telestroke services in US EDs. For both applications, the most frequently reported single most important reason for adoption was related to improving clinical care. Notably, EDs with pediatric telehealth services used the technology much less frequently than EDs with telestroke services. There may be value for smaller EDs to benefit from economies of scale in telehealth implementation in order to address disparities in access to care.

## Appendix

Multimedia Appendix 1 The 2016 National Emergency Department Inventory–USA (NEDI-USA), example follow-up survey, and supplemental tables.

## Abbreviations

DRG: diagnosis-related group
ED: emergency department
EMNet: Emergency Medicine Network
EMS: emergency medical services
NEDI-USA: National Emergency Department Inventory–USA
REDCap: Research Electronic Data Capture
telestroke: telehealth for emergency stroke care delivery
