# Supplementary material for: Emergency Departments’ Uptake of Telehealth for Stroke Versus Pediatric Care: Observational Study
Source: J Med Internet Res. 2022 Jun 20;24(6):e33981. doi: 10.2196/33981 (PMC9254043; doi:10.2196/33981)
Supplement: Multimedia Appendix 1 [file jmir_v24i6e33981_app1.docx]

**Multimedia Appendix 1**

**NEDI-USA 2016**

**
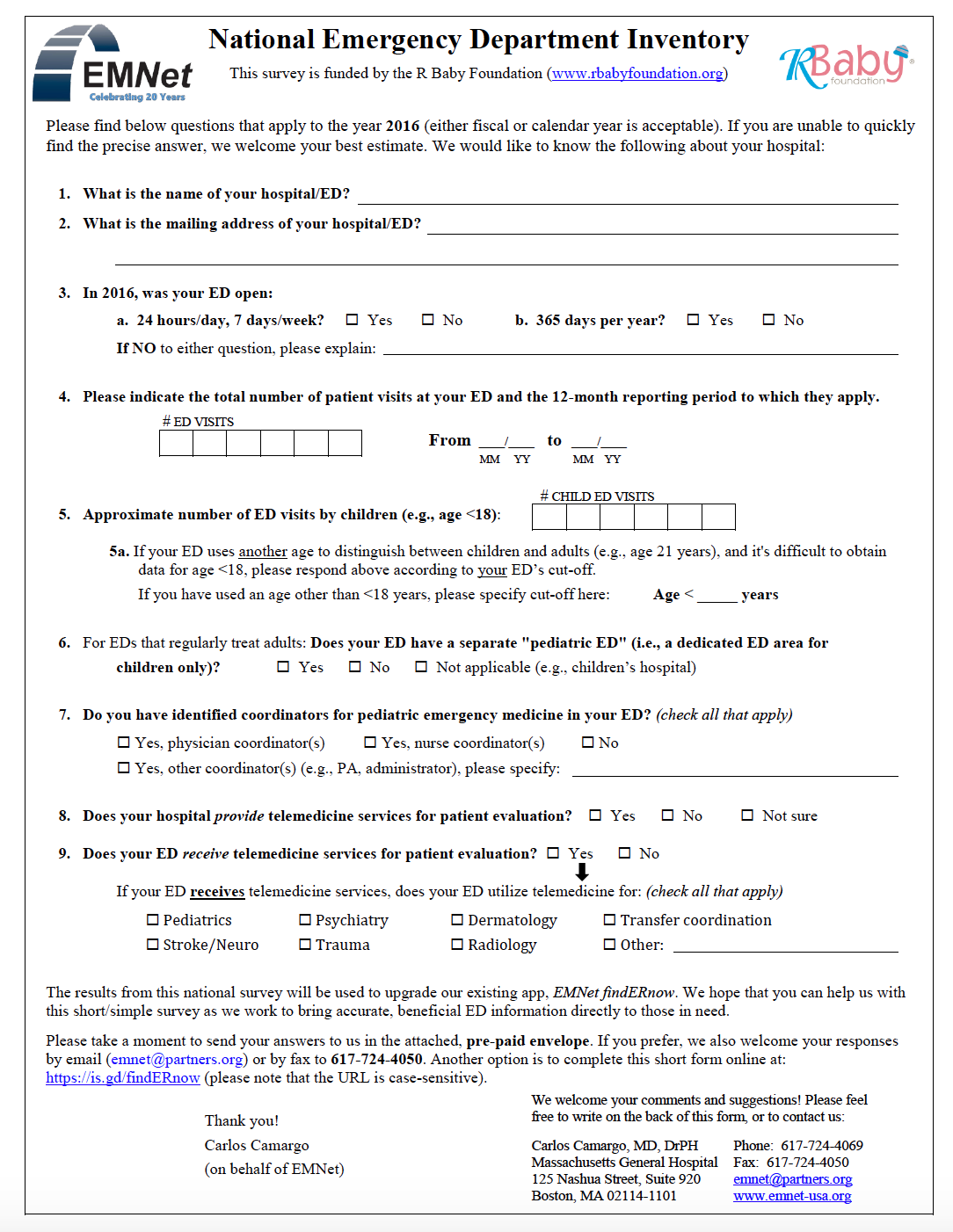
**

**Example of follow-up survey**


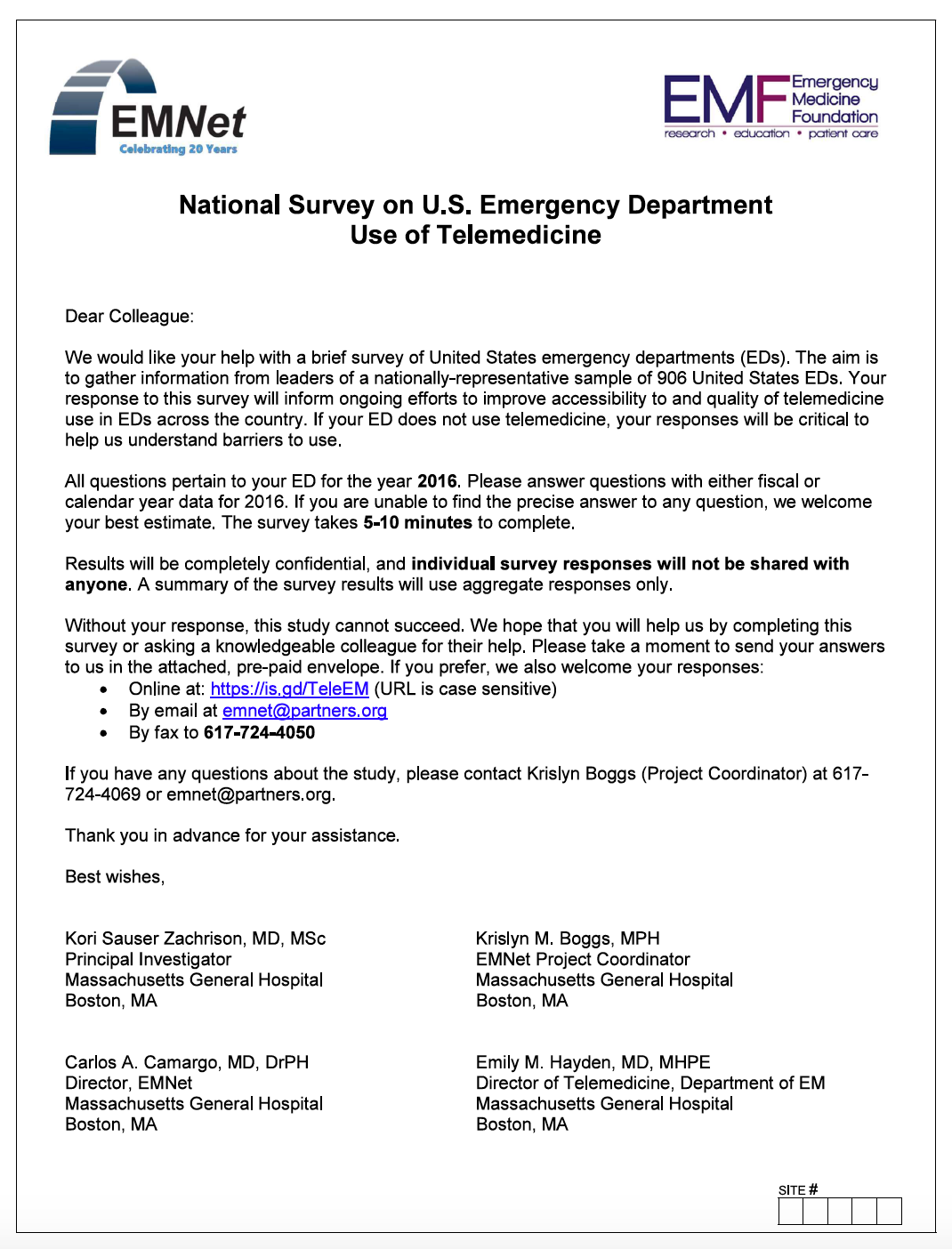


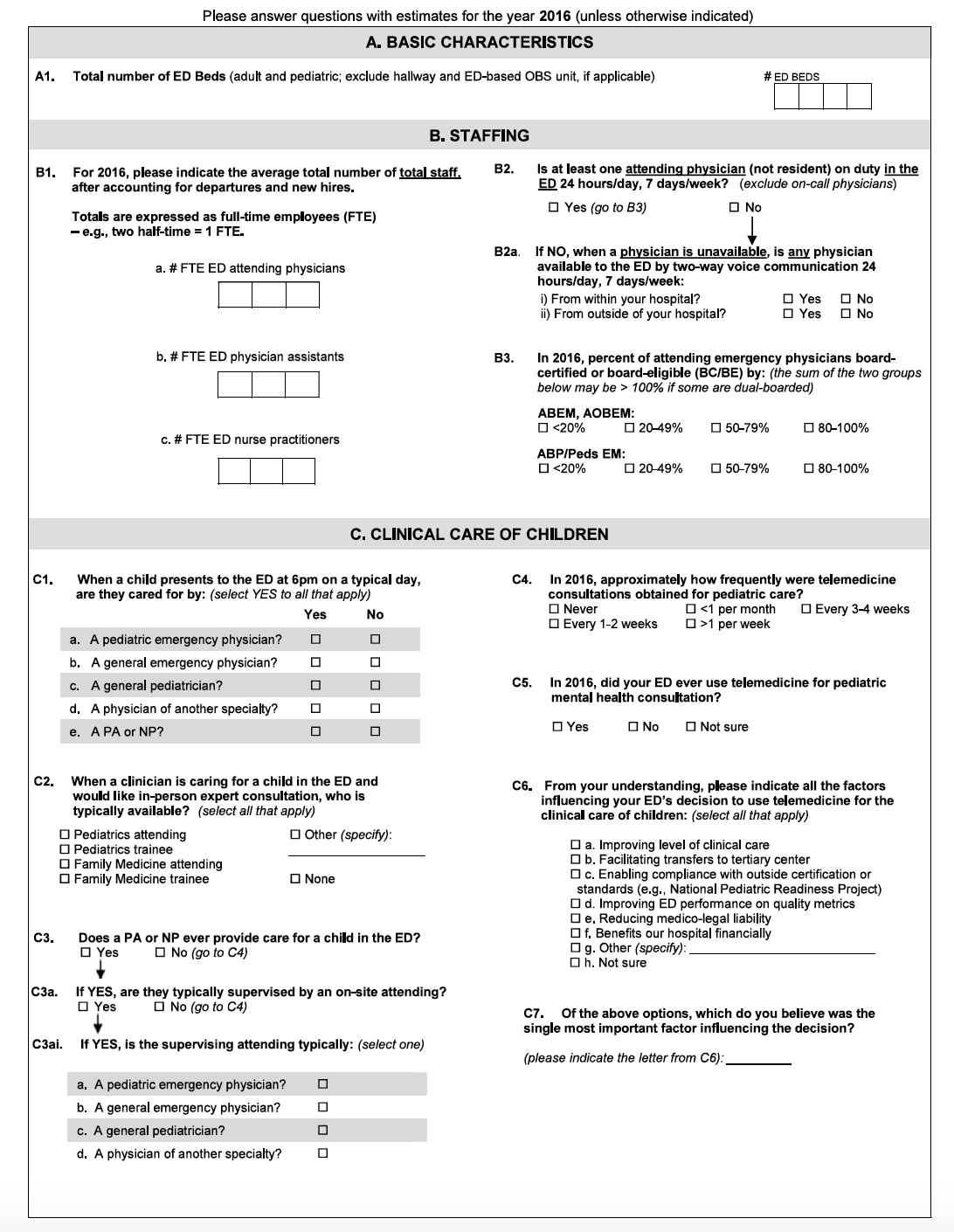


**
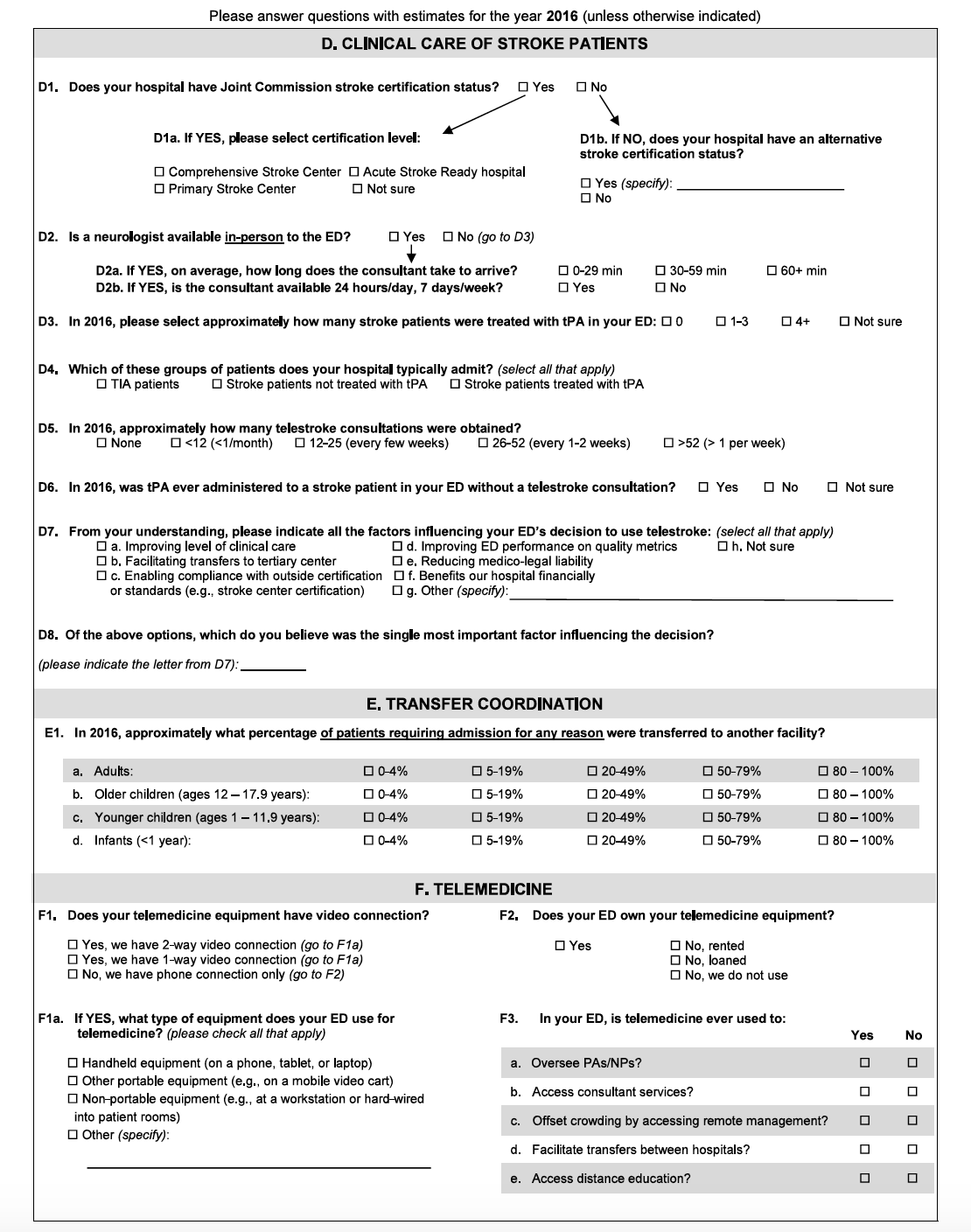
**

**
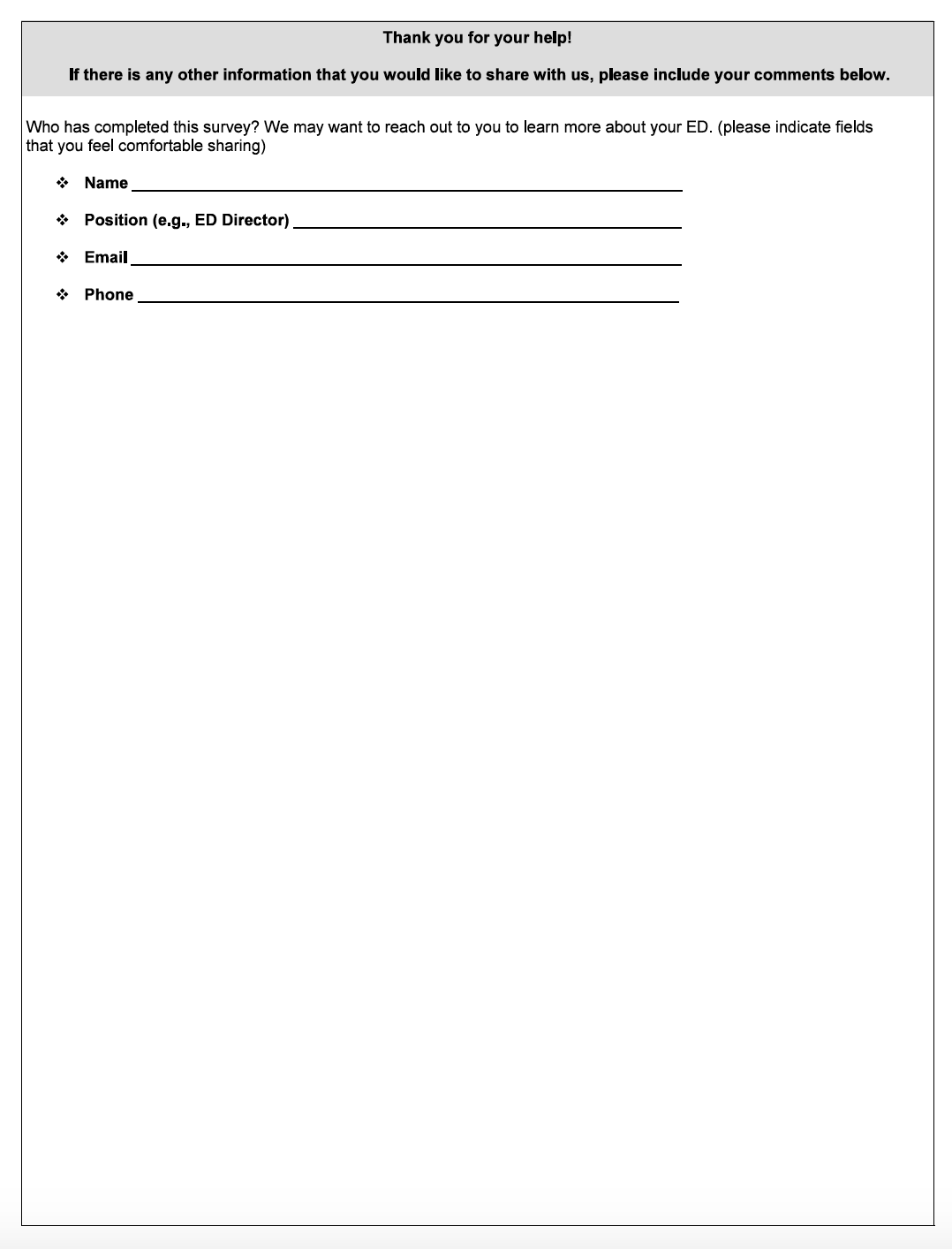
**

**Table S1. Among EDs indicating ‘Other’ as reason for adoption of telestroke, classification of free-responses**

|  | Policy-motivated | Not policy-motivated |
| --- | --- | --- |
| Selecting ‘Other’ for reason for Pediatric TM adoption  (n = 20) |  | Faculty location  We had no specialists available  Higher level of care  Specialist consult  Assessing burn patients  Did not use it though it was available  Don’t take peds in house  Language barriers  Pedi services not available  Reassurance to nursing staff  Specialty case services needed  Access to specialists  At request of larger facility to facilitate appts  Better pt outcome  Distance to childrens hospital  Do not use  Provide the experience of tertiary Ped EM to our ERs  Psych  Rare, so good resource  We need pediatrics specialty because we’re a rural facility |
| Selecting ‘Other’ for reason for telestroke adoption  (n = 27) |  | Help with decision to give tPA  Availability of more neurologists  Decision making support  Distance to another facility  Do not use: have in house neurology  Free program  Lack of neurology availability  Make stroke care available when stroke consult is unavailable  N/A Do not use  NA  Neurology not available 24/7 on site  No Neuro Coverage- Staffing  No in-person neurologist  XXX is Stroke Certified. We are a small sister hospital. Provider coverage when staff neurologist cannot take call.  Provider not want to use it.  Trouble keeping neurologists employed  Unavailability of services  Volume in ED and only a few neurologist consults available  At one point didn’t have a physician on call  Better pt outcome  Decisions to admit or transfer  Do not use  No neurologist available for consults in our hospital  No telestroke-have tele-emergency care  We do not use telestroke in our ED  We don’t use telehealth for telestroke care |

**Table S2. Characteristics of Non-responding versus Responding Emergency Departments to Telehealth Survey, n=915**

|  | Non-responders to TM survey (N=148) | Responded to TM survey (N=767) |
| --- | --- | --- |
| ED Characteristics | n (%) | n (%) |
| Received telehealth services for patient evaluation in 2016 | 77 (52) | 385 (50) |
| Total visit volume in 2016, median (IQR) | 12,290 (6,173-32,066) | 6,614 (2,298-14,600) |
| Pediatric visits volume in 2016, median (IQR) | 2,209 (1,023-4,782) | 1,293 (328-3,000) |
| Rural location (CBSA) | 85 (57) | 548 (71) |
| Region |  |  |
| Northeast | 10 (7) | 66 (9) |
| Midwest | 32 (22) | 299 (39) |
| South | 69 (47) | 259 (34) |
| West | 37 (25) | 143 (19) |
| Freestanding | 5 (3) | 22 (3) |
| Critical access hospital | 69 (47) | 476 (62) |
| Academic | 1 (1) | 7 (1) |
| Pediatric Emergency Care Coordinator | 28 (19) | 73 (10) |
